# A Novel Heterocyclic Compound CE-104 Enhances Spatial Working Memory in the Radial Arm Maze in Rats and Modulates the Dopaminergic System

**DOI:** 10.3389/fnbeh.2016.00020

**Published:** 2016-02-22

**Authors:** Yogesh D. Aher, Saraswathi Subramaniyan, Bharanidharan Shanmugasundaram, Ajinkya Sase, Sivaprakasam R. Saroja, Marion Holy, Harald Höger, Tetyana Beryozkina, Harald H. Sitte, Johann J. Leban, Gert Lubec

**Affiliations:** ^1^Department of Pediatrics, Medical University of ViennaVienna, Austria; ^2^Institute of Pharmacology, Center for Physiology and Pharmacology, Medical University of ViennaVienna, Austria; ^3^Core Unit of Biomedical Research, Division of Laboratory Animal Science and Genetics, Medical University of ViennaHimberg, Austria; ^4^Ural Federal UniversityYekaterinburg, Russia; ^5^Department of Pharmaceutical Chemistry, University of ViennaVienna, Austria

**Keywords:** CE-104, cognitive enhancement, dopamine uptake inhibitor, monoamine neurotransmitter uptake inhibitor, radial arm maze, dopamine receptor complexes, dopamine transporter complexes

## Abstract

Various psychostimulants targeting monoamine neurotransmitter transporters (MATs) have been shown to rescue cognition in patients with neurological disorders and improve cognitive abilities in healthy subjects at low doses. Here, we examined the effects upon cognition of a chemically synthesized novel MAT inhibiting compound 2-(benzhydrylsulfinylmethyl)-4-methylthiazole (named as CE-104). The efficacy of CE-104 in blocking MAT [dopamine transporter (DAT), serotonin transporter (SERT), and norepinephrine transporter] was determined using *in vitro* neurotransmitter uptake assay. The effect of the drug at low doses (1 and 10 mg/kg) on spatial memory was studied in male rats in the radial arm maze (RAM). Furthermore, the dopamine receptor and transporter complex levels of frontal cortex (FC) tissue of trained and untrained animals treated either with the drug or vehicle were quantified on blue native PAGE (BN-PAGE). The drug inhibited dopamine (IC_50_: 27.88 μM) and norepinephrine uptake (IC_50_: 160.40 μM), but had a negligible effect on SERT. In the RAM, both drug-dose groups improved spatial working memory during the performance phase of RAM as compared to vehicle. BN-PAGE Western blot quantification of dopamine receptor and transporter complexes revealed that D1, D2, D3, and DAT complexes were modulated due to training and by drug effects. The drug’s ability to block DAT and its influence on DAT and receptor complex levels in the FC is proposed as a possible mechanism for the observed learning and memory enhancement in the RAM.

## Introduction

Psychostimulants are a class of psychoactive compounds that have diverse behavioral effects such as increasing arousal, alertness and motivation, altering mood, facilitating working, and long term memories. It can improve cognition in patients with cognitive disabilities caused by various psychiatric illnesses like Alzheimer disease, attention deficit hyperactivity disorder (ADHD), or schizophrenia (Goodman and Gilman, 2006; Berridge and Devilbiss, 2011). Basic research and clinical trials show that these compounds improve performance in cognitive challenging tasks also in healthy subjects (Husain and Mehta, 2011).

The majority of these compounds inhibit monoamine neurotransmitter transporters (MATs) which reuptake the released neurotransmitters back into the presynaptic nerve terminal (Kristensen et al., 2011), thus induce an increase of extracellular monoamine neurotransmitter (MNT) levels and related subsequent signaling (Berridge and Devilbiss, 2011). Moreover, this class of drug specifically targets MAT of prefrontal cortex (PFC) regions, which are largely implicated in mediating complex executive cognitive functions (Spencer et al., 2012). The resulting elevated levels of monoamines in the PFC are associated with improved cognition (Berridge and Devilbiss, 2011). Other mechanism includes activation of MNT receptors or candidate targets of the downstream signaling pathway, increasing the oxygen and glucose supply to the nerve cells (Froestl et al., 2012, 2014a,b).

A number of MAT inhibitors have been documented to be effective in various cognitive tasks. For instance, modafinil that inhibits dopamine reuptake in the synapse has been shown to improve spatial memory in the multiple T-maze (Sase et al., 2012) and in the Morris water maze (Shuman et al., 2009; Tsanov et al., 2010). However, the actions of modafinil are diverse in different learning paradigms. It specifically improves the working memory but not the spatial reference memory in rats tested in radial arm maze (RAM; Sase et al., 2012). In delayed non-matching to position task in the water maze, 55 and 100 mg/kg made significantly more correct choices, the latter said dose enhanced the locomotor activity, but with no efficient goal-directed behavior in the rats (Ward et al., 2004). In spatial serial discrimination (reversal task; Béracochéa et al., 2002, 2003) and in delayed spontaneous alteration in T-maze (Béracochéa et al., 2001) modafinil had effect only in high dose of 64 mg/kg but had no effect in relatively low clinically relevant doses. In contextual serial discrimination task performed in mice using hole board apparatus, modafinil 32 but not a 16 mg/kg dose enhanced the successful discriminations, whereas it had no effect in spatial serial discrimination task (Béracochéa et al., 2008). In other paradigms, five-choice serial reaction time task, that tests the attention (Waters et al., 2005) and in object recognition task (Garcia et al., 2013) modafinil did not had any effects in any of the doses tested.

Amphetamines which are a diverse group of compounds that block MAT with different specificities are distinct in their mode of action compared to other psychostimulants (Fleckenstein et al., 2007). Although competitive inhibitors of MAT, they can exert their actions in other ways as (a) they are taken up by the plasma-membrane MAT as exogenous substrates (Sitte et al., 1998), (b) induce eﬄux of monoamines by aiding the release of neurotransmitter vesicles (Partilla et al., 2006), and (c) act by MAT reverse transport mechanism (Robertson et al., 2009). The latter said effect is predominant in magnitude in amphetamine’s psychostimulant effects (Sitte and Freissmuth, 2015). In doses less than 0.05 mg/kg in rats, amphetamine enhanced the freezing reaction in a cued fear conditioning paradigm whereas at the same dose it had no effect on a contextual fear conditioning test. In contrast, modafinil had opposite actions in fear conditioning tests, increasing contextual memory whereas cued related memory remains unaffected (Shuman et al., 2009; Shanmugasundaram et al., 2015a). Thus, these drugs can produce different effects in learning and memory tasks based on the nature of cognitive tasks, the dosage, MAT blocking profile and activation of drug specific various other cellular targets.

In order to overcome these heterogeneous effects, to eliminate abuse potentials and also to reduce the adverse effects, attempts are still being made to develop novel drugs targeting MAT to increase the effects upon cognitive function in healthy subjects and to treat mental disorders. New drugs should also meet the demands of increased specificity of the effects on cognitive performance possibly with no or least side effects. Therefore, a novel heterocyclic compound was chemically synthesized by replacing the amide group of modafinil with a heterocyclic group. The MNT reuptake blocking capability was studied using *in vitro* uptake assays and effects on cognition were tested in rats in a RAM spatial memory paradigm. The task demands the establishment of a complex learning strategy involving WM and the PFC to successfully find the reward with minimal efforts (Vertes, 2006). Further, the dopamine receptor complexes (RCs) and total DAT as well as phosphorylated DAT (DAT-ph) complex levels in the frontal cortex (FC) tissue of animals trained in the RAM with drug or vehicle were also quantified and compared with controls.

## Materials and Methods

### Chemical Synthesis of CE-104

CE-104 was chemically synthesized by following a modified procedure used for the synthesis of modafinil (Courvoisier et al., 2012). The general scheme of the synthesis is shown in Supplementary Figure S1. The structure representing modafinil was converted to appropriate thioamide by Lawesson’s reagent. The obtained product was converted to the methylthiazole analog with chloromethyl ketone in Hantsch-like synthesis. The final compound was obtained in the racemic form by oxidation of sulfur with 30% H_2_O_2_ in glacial acetic acid.

The step by step synthesis procedure is described in the Supplementary Material.

### Monoamine Neurotransmitter Uptake Assay

The following materials were purchased from companies; Dulbecco’s modified Eagle’s medium (DMEM) and trypsin from PAA Laboratories GmbH (Pasching, Austria). Fetal bovine serum was from Invitrogen. [^3^H]5-HT ([^3^H]5-hydroxytryptamine; [^3^H]serotonin; 28.3 μCi/mmol) and [^3^H]DA ([^3^H]dihydroxyphenylethylamine; [^3^H]dopamine; 46 μCi/mmol) were from Perkin Elmer, Boston, MA, USA. [^3^H]1-Methyl-4-phenylpyridinium ([^3^H]MPP+; 85 μCi/mmol) was supplied by American Radiolabeled Chemicals (St. Louis, MO, USA). Paroxetine from Santa Cruz Biotechnology, USA, and mazindole and D-amphetamine from Sigma-Aldrich, Co.

HEK293 cells stably expressing human isoforms of DAT, serotonin transporter (SERT) and norepinephrine transporter (NET) named as HEK-DAT, HEK-SERT, and HEK-NET respectively were used for this purpose. Effects of CE-104 on uptake of their respective substrates were analyzed as described by Sucic et al. (2010). In brief, the cells were grown in poly-d-lysine coated 96-well plates in DMEM containing 10% fetal bovine serum. CE-104 was dissolved first in 100% dimethyl sulfoxide (DMSO) and subsequently diluted in Krebs–Ringer–HEPES buffer (KHB; 25 mM HEPES.NaOH, pH 7.4, 120 mM NaCl, 5 mM KCl, 1.2 mM CaCl_2_, and 1.2 mM MgSO_4_ supplemented with 5 mM D-glucose). To determine unspecific uptake, 10 μM of mazindole were used in HEK-DAT and HEK-NET cells and 10 μM of paroxetine was used for HEK-SERT. The titrated substrates used to assess transport activity in HEK-DAT, HEK-SERT, and HEK-NET were 0.2 μM [^3^H]DA, 0.4 μM [^3^H]5HT, and 0.05 μM [^3^H]MPP^+^, respectively. The cells were washed once with KHB buffer and incubated with CE-104 either 5 min for HEK-DAT and HEK-SERT cells or 8 min for HEK-NET cells. Subsequently, the substrates were added and incubated for 1 min for HEK-DAT and HEK-SERT cells or 3 min for HEK-NET cells and reactions were stopped with ice-cold KHB buffer. The cells were harvested using trypsin and lysed with 1% SDS and released radioactivity was measured by a liquid scintillation counter (Tri-carb-2300TR, Perkin Elmer).

### Radial Arm Maze

#### Animals

Seventy two male Sprague Dawley rats, aged between 12 and 14 weeks, were used for the experiments. They were bred and maintained in cages made of Makrolon filled with autoclaved woodchips in the Core Unit of Biomedical Research, Division of Laboratory Animal Science and Genetics, Medical University of Vienna. Food and water in bottles were available *ad libitum* and reared under normal animal facility conditions (temperature: 22 ± 2°C; humidity: 55 ± 5%; 12 h artificial light/12 h dark cycle: light on at 7:00 am). The behavioral experiments were conducted between 8:00 am and 2:00 pm. All procedures were carried out according to the guidelines of the Ethics committee, Medical University of Vienna, and were approved by Federal Ministry of Education, Science and Culture, Austria (BMWFW-66.009/0114-WF/II/3b/2014).

#### Apparatus

The RAM setup is made of black plastic and kept at an elevation of 80 cm above the floor in a room with numerous visual cues placed distally on all sides. The central platform had a diameter of 50 cm with 12 arms (12 cm × 60 cm) projecting radially outward. A plastic cylinder placed in the center was used to restrict the movement of rats before the start of the training. The lifting of the cylinder is controlled by a pulley system from the far end of the room. Food is always placed 5 cm from the distal end of the arm. The training sessions were recorded with a computerized tracking video camcorder (1/3 SSAM HR EX VIEW HAD).

#### Drug Administration

CE-104 was freshly dissolved in DMSO and injected everyday 30 min before the start of the behavior experiment throughout the training and performance sessions. Pure DMSO was used as vehicle. Rats received 1 ml/kg drug or vehicle administered intraperitoneally in doses of 1 and 10 mg/kg body weight.

#### The Training Procedure

Radial arm maze training was performed using a modified protocol as described by Levin et al. (2010). The amount of food provided was restricted for 5 days prior to the experiment to reduce the body weight to 85% to motivate the rats for foraging behavior during the training sessions. Water was provided *ad libitum* throughout the entire training procedure. The rats were handled for 30 min/day during these 5 days for adaptation to the experimenter. Two days before the start of the actual training, rats were given habituation session for 5 min in which some food pellets were placed scattered all over the maze and rats were allowed to explore the maze and let consume the food. During the training sessions, out of 12 arms, eight arms were baited only once at the beginning of each session and the remaining four were kept unbaited. The pattern of baited and unbaited arms remained unchanged throughout the entire procedure for each rat but differed among rats. Each trial began by placing the rat in the central platform, after 10 s the cylinder was slowly lifted. A session lasts 8 min or until all eight baited arms were entered. The rats were given one training session per day over a period of 10 days. The first entry into an unbaited arm was noted as a reference memory error (RME), second and subsequent entries into unbaited arms were counted as a working memory error (WME-unbaited), second and subsequent entry into the baited arm was noted as working memory error (WME-baited). The time taken to complete a trial was also recorded as latency.

Untrained animals (yoked) were exposed to the maze and spatial cues for same amount of time as their trained counterparts but without reward, therefore rats did not learn to associate the cues with the reward.

### Blue Native-Polyacrylamide Gel Electrophoresis (BN-PAGE)

Six hours after the last session of RAM training on day 10, the animals were sacrificed after anaesthetizing them with CO_2_. The brain was quickly extracted and was kept on a cold plate and the FC was cut 3 mm from the anterior end after removing olfactory bulb by a chilled razor blade. The tissues were immediately frozen in dry ice and were stored at –80°C. Only the 10 mg/kg dose group was used for biochemical studies.

#### Sample Preparation

Crude synaptosome fractions were prepared from the FC as described previously (Shanmugasundaram et al., 2015b). The samples were maintained at 4°C throughout the procedure. In brief, the FC tissues were homogenized in 5 ml of ice cold homogenization buffer containing 10 mM HEPES, pH 7.7, 300 mM sucrose, 1 mM EDTA, protease inhibitor cocktail (Roche Diagnostics, Mannheim, Germany) using Ultra-Turrax^®^ (IKA, Staufen, Germany). The homogenate was centrifuged for 10 min at 1000 × *g* and the pellet was discarded. The supernatant was centrifuged at 50,000 × *g* for 30 min using an ultracentrifuge (Beckman Coulter Optima-L-90K). The pellet was re-suspended in washing buffer (homogenization buffer without sucrose), kept on ice for 1 h and centrifuged at 50,000 × *g* for 30 min to obtain membrane fractions of crude synaptosome extracts.

The synaptosome membrane pellets were solubilized in extraction buffer (1.5 M 6-aminocaproic acid, 300 mM Bis-Tris, pH 7.0) containing 1% Triton X-100 with vortexing every 10 min for 1 h. Following solubilization, samples were cleared by centrifugation at 20,000 × *g* for 60 min at 4°C. The protein content of the supernatant was estimated using the BCA protein assay kit (Pierce, Rockford, IL, USA). Extracted proteins were then aliquoted and stored at –80°C.

#### BN-PAGE Western Blot Procedure

Equal amounts of proteins (70 μg) from all groups were loaded in the wells and the RCs were separated on 5–13% of blue native PAGE gels and the Western blot procedure was carried out using the procedure described previously (Kang et al., 2009). The details of antibodies used are as follows; D1R (1:5000, Abcam-ab81296, Cambridge, UK), D2R (1:5000, Abcam-ab21218, Cambridge, UK), D3R (1:5000, Abcam-ab42114, Cambridge, UK), pDAT (1:5000, DAT Thr53, Phosphosolutions-p435-53, Aurora, CO, USA) and DAT (1:5000, Abcam- ab111468, Cambridge, UK) and detected with horseradish peroxidase-conjugated anti-rabbit IgG secondary antibody (1:10000, Abcam-ab6721, Cambridge, UK). Immunoreactive bands were quantified by the software Image J (NIH). Coomassie blue R-350 stained membranes were used as loading control and normalized with the Western blot densitometric values (Welinder and Ekblad, 2011; Sase et al., 2012).

### Statistical Analysis

Non-linear regression analysis was carried out to determine the IC_50_ values for uptake assays. The effect of training and the effect of drug on learning performance in RAM were analyzed using two-way ANOVA (with Bonferroni adjustments) with drug effects and training effects as the two factors performed on the dependent variables WME-baited, WME-unbaited, RME and latency. For day-by-day analysis to compare the drug effects, pairwise multiple comparison was done using univariate analysis with LSD *post hoc* test. The densitometry values of BN-PAGE Western blot quantification were compared using two-way ANOVA (training X drug) with LSD *post hoc* test for multiple comparison to reveal the differences between groups vehicle-trained, CE-104-trained, vehicle-yoked and CE-104-yoked. The probability level of *p* < 0.05 was considered as statistically significant. Regression analysis was done using GraphPad Prism (version 6.00, GraphPad Software, San Diego CA, USA). ANOVA was performed using SPSS (version 20.0; SPSS, Inc., Chicago, IL, USA).

## Results

### CE-104 Blocks DAT with Modest and NET with Lower Affinity

**FIGURE 1:**
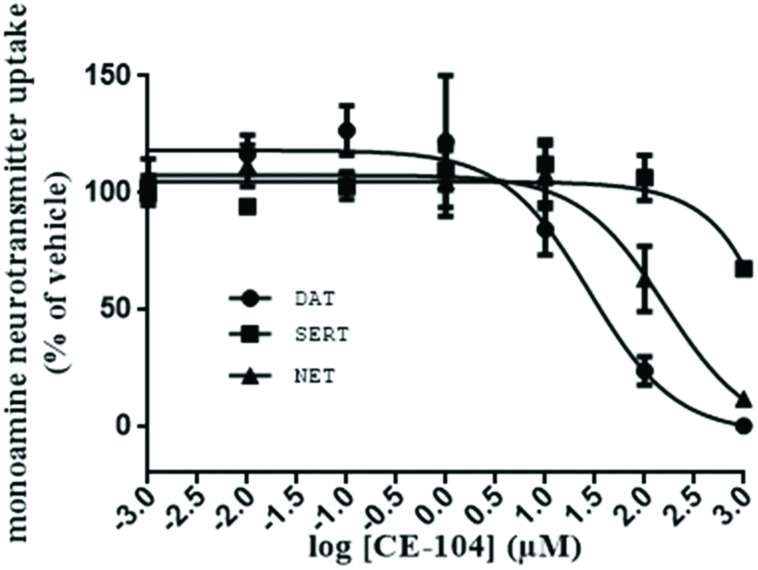
**Evaluation of monoamine neurotransmitter transporter blockade of CE-104: inhibition of dopamine and norepinephrine transport by CE-104 in HEK293 cells stably expressing human isoforms of DAT, SERT, and NET.** CE-104 inhibited dopamine uptake with IC_50_ of 27.88 ± 0.104 μM (*n* = 9) and norepinephrine uptake with IC_50_ of 160.40 ± 0.09561 μM (*n* = 9). Inhibition of SERT was negligible (IC_50_: ∼955009 ± 440.4 μM).

The efficacy of CE-104 to block the DAT, SERT, and NET from the uptake of their respective substrates [^3^H]DA, [^3^H]5HT, and [^3^H]MPP^+^ was determined. **Figure 1** shows that CE-104 blocks DAT with IC_50_ = 27.88 ± 0.104 μM (*n* = 9) while the effect on NET was IC_50_ = 160.40 ± 0.09561 μM (*n* = 9). In case of SERT the CE-104 blockade effects were negligible (IC_50_: ∼955009 ± 440.4 μM; *n* = 9).

### CE-104 Improves Working Memory Performance in RAM

**FIGURE 2:**
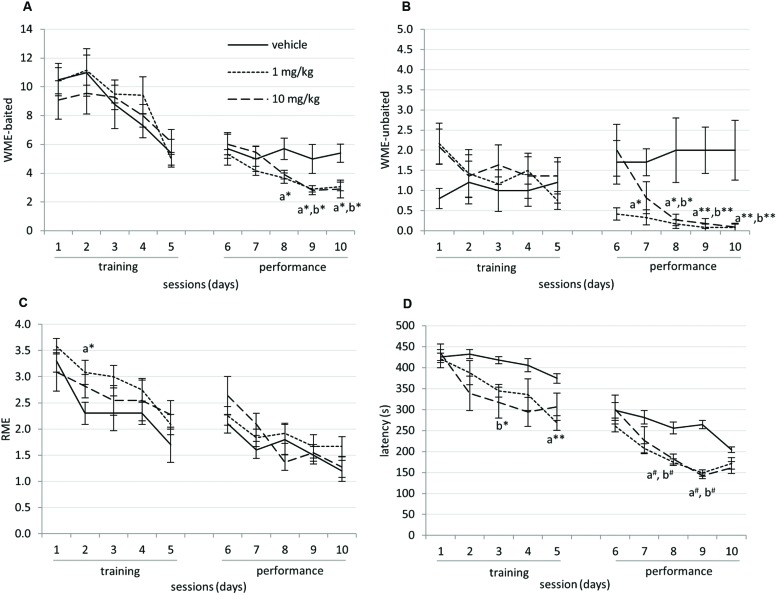
**(A)** Working memory error (WME)-baited reduced significantly due to repeated training (*p* < 0.0001) and were significantly decreased in drug treated group on days-8, 9, and 10 compared to vehicle. **(B)** WME-unbaited were significantly decreased in drug treated group on days-7, 8, 9, and 10 compared to vehicle. **(C)** RME (Reference memory error) reduced significantly with the respect to training sessions (*p* < 0.01). **(D)** Latency to complete the task declined significantly during the training and performance sessions (*p* < 0.001). Mean and SEM are shown in the graph. The data were analyzed using two-way ANOVA with Bonferroni *post hoc* adjustments for within subject effects. Day-by-day comparison was performed using univariate analysis with LSD adjustments. x – vehicle vs. 1 mg/kg, y – vehicle vs. 10 mg/kg (^∗^*p* < 0.05; ^∗∗^*p* < 0.01; #*p* < 0.005).

The total number of WME-baited, WME-unbaited, RME and latency to finish a session by the animals during the training and performance sessions were shown in **Figure 2.** In the two-way ANOVA analysis, due to the effect of training the vehicle, 1 and 10 mg/kg groups showed significant decrease of WME-baited during the learning *F*(4,145) = 9.823, *p* < 0.0001 and in the performance sessions *F*(4,145) = 5.927, *p* < 0.0001. The RME significantly declined in the training phase *F*(2,150) = 4.867, *p* < 0.005 and in the performance phase *F*(2,145) = 21.756, *p* < 0.0001. The latency to finish a session was also declined significantly during the learning *F*(4,150) = 7.762, *p* < 0.001 and in the performance phase *F*(4,150) = 21.645, *p* < 0.001. The drug treatment had significant effect on RME, during the training phase *F*(4,150) = 9.482, *p* < 0.0001, WME-baited during the performance phase *F*(2,145) = 8.848, *p* < 0.0001, WME-unbaited during the performance phase *F*(2,145) = 21.756, *p* < 0.0001 and latency during the training *F*(2,150) = 10.585, *p* < 0.001 and the performance phase *F*(2,150) = 23.985, *p* < 0.001.

Day-by-day analysis comparing vehicle and drug treated groups revealed that in the performance phase of RAM, WME-baited were significantly decreased in both 1 and 10 mg/kg drug treated groups on day-8 (vehicle vs. 1 mg/kg: *p* < 0.05), day-9 (vehicle vs. 1 mg/kg: *p* < 0.05, vehicle vs. 10 mg/kg: *p* < 0.05), and day-10 (vehicle vs. 1 mg/kg: *p* = 0.018, vehicle vs. 10 mg/kg: *p* < 0.05). WME-unbaited significantly decreased in both, 1 and 10 mg/kg drug treated groups on day-7 (vehicle vs. 1 mg/kg: *p* < 0.05), day-8 (vehicle vs. 1 mg/kg: *p* < 0.05, vehicle vs. 10 mg/kg: *p* < 0.05), day-9 (vehicle vs. 1 mg/kg: *p* < 0.005, vehicle vs. 10 mg/kg: *p* < 0.005), and day-10 (vehicle vs. 1 mg/kg: *p* < 0.01, vehicle vs. 10 mg/kg: *p* < 0.01) compared to vehicle. RME increased in 1 mg/kg group on day-2 (vehicle vs. 1 mg/kg, *p* < 0.05). The latency decreased significantly on day-3 (vehicle vs. 10 mg/kg: *p* < 0.05) and day-5 (vehicle vs. 1 mg/kg: *p* < 0.01) in the training session and also during performance session on day-8 (vehicle vs. 1 mg/kg: *p* < 0.005) and on day-9 (vehicle vs. 10 mg/kg: *p* < 0.005).

The F ratios and p values of the one way repeated measure ANOVA statistical analysis was shown in the **Table 1.**

**Table 1:** Showing the statistical *F* ratios and *p* values of two way ANOVA analysis of training and performance sessions of RAM.

Independent variables	Learning sessions	Effect of training	Effect of drug treatment		
				Multiple comparison	
				Vehicle vs. 1 mg/kg	Vehicle vs. 10 mg/kg
WME-baited	Training	*F*(4,145) = 9.823, *p* < 0.0001	*F*(2,145) = 0.692, *p* = 0.502	–	–
	Performance	*F*(4,145) = 5.927, *p* < 0.0001	*F*(2,145) = 8.848, *p* < 0.0001	*p* < 0.0001	*p* < 0.05
WME-unbaited	Training	*F*(4,145) = 0.532, *p* = 0.712	*F*(2,145) = 1.965, *p* = 0.144	–	–
	Performance	*F*(4,145) = 1.192, *p* = 0.317	*F*(2,145) = 21.756, *p* < 0.0001	*p* < 0.0001	*p* < 0.0001
RME	Training	*F*(2,150) = 4.867, *p* < 0.005	*F*(4,150) = 9.482, *p* < 0.0001	*p* < 0.01	–
	Performance	*F*(2,145) = 21.756, *p* < 0.0001	*F*(4,145) = 1.192, *p* = 0.317	–	–
Latency	Training	*F*(4,150) = 7.762, *p* < 0.001	*F*(2,150) = 10.585, *p* < 0.001	*p* < 0.001	*p* < 0.001
	Performance	*F*(4,150) = 21.645, *p* < 0.001	*F*(2,150) = 23.985, *p* < 0.001	*p* < 0.001	*p* < 0.001

### Training and CE-104 Modulates Synaptic Dopamine Receptors D1, D2, and D3 Levels

**FIGURE 3:**
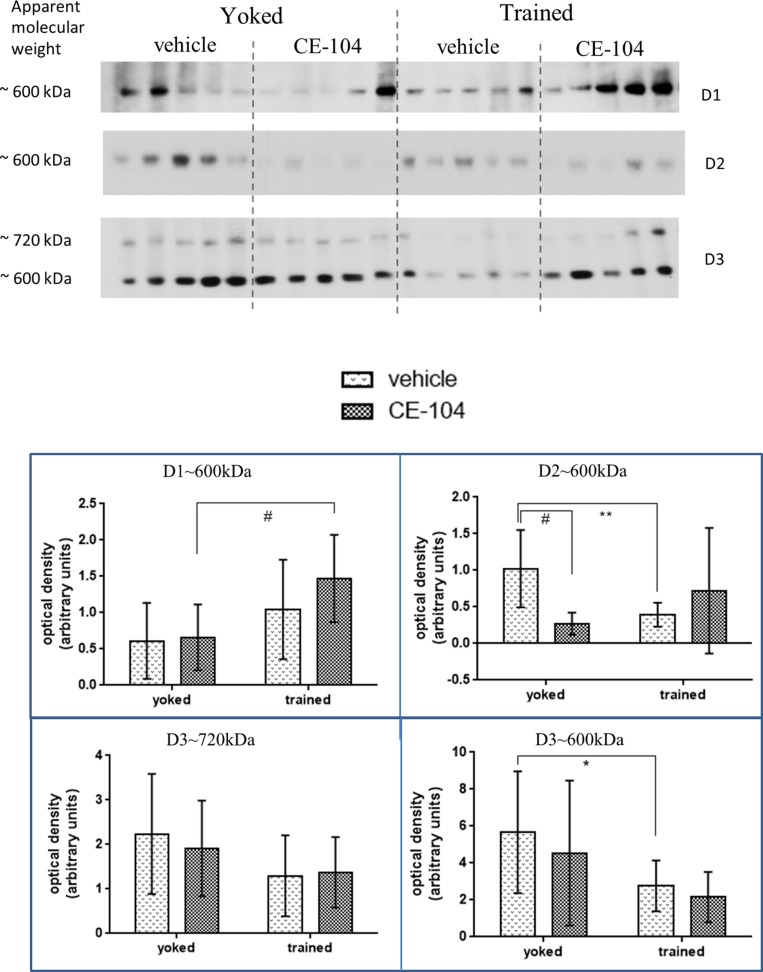
**Representative blot of BN-PAGE Western quantification of dopamine receptor complexes in frontal cortex tissue.** D1∼600 kDa, D2∼600 kDa, and D3∼600 kDa receptor complexes were modulated as a result of training and drug treatment. Graph shows mean ± SD. Special characters indicate level of significance of difference in two-way ANOVA (^∗^*p* < 0.05; ^∗∗^*p* < 0.01; #*p* < 0.005).

Two-way ANOVA analysis of BN-PAGE Western blot densitometric values of dopamine RCs were shown in **Figure 3.** Multiple comparisons using LSD *post hoc* test revealed differences between the groups.

For the D1 receptor which was separated at 600 kDa: in drug injected groups, D1∼600 kDa containing complex levels were increased significantly in trained groups compared to yoked groups (*p* < 0.005) whereas there was no significant change in vehicle groups. For the D2 receptor which was separated at 600 kDa: in the vehicle treated group D2∼600 kDa containing complex levels were significantly lowered in trained animals (*p* < 0.01) whereas, there were no changes in the drug-treated group and the levels were significantly decreased in drug-treated animals compared to vehicle in yoked groups (*p* < 0.01). For the D3 receptor which was separated at 600 kDa: in vehicle treated groups D3∼600 kDa containing complex levels were decreased significantly in trained animals compared to the yoked groups (*p* < 0.05).

The *F* ratios and *p* values of the two way repeated measure ANOVA statistical analysis were shown in the **Table 2.**

**Table 2:** Showing the statistical *F* ratios and *p* values of the two way ANOVA of the Western blot quantification of the receptor complexes in BN-PAGE.

Receptor complex	Training effect	Drug effect	Interaction
D1R ∼600 kDa	*F*(1,36) = 11.76, *p* < 0.005	*F*(1,36) = 1.731, *p* = 0.1966	*F*(1,36) = 1.101, *p* = 0.3011
D2R ∼720 kDa	*F*(1,35) = 0.03396, *p* = 0.8549	*F*(1,35) = 3.494, *p* = 0.0700	*F*(1,35) = 2.822, *p* = 0.1019
D2R ∼600 kDa	*F*(1,35) = 0.3112, *p* = 0.5805	*F*(1,35) = 1.722, *p* = 0.1979	*F*(1,35) = 11.27, *p* < 0.005
D3R ∼720 kDa	*F*(1,36) = 4.926, *p* < 0.05	*F*(1,36) = 0.1426, *p* = 0.7080	*F*(1,36) = 0.3582, *p* = 0.5532
D3R ∼600 kDa	*F*(1,35) = 8.868, *p* < 0.001	*F*(1,35) = 0.9438, *p* = 0.3380	*F*(1,35) = 0.07993, *p* = 0.7791
DAT ∼720 kDa	*F*(1,36) = 1.777, *p* = 0.1909	*F*(1,36) = 4.794, *p* < 0.05	*F*(1,36) = 1.718, *p* = 0.1982
DAT ∼600 kDa	*F*(1,35) = 5.487, *p* < 0.05	*F*(1,35) = 0.2377, *p* = 0.6289	*F*(1,35) = 0.9620, *p* = 0.3334
DAT-ph ∼720 kDa	*F*(1,35) = 1.802, *p* = 0.1881	*F*(1,35) = 0.3607, *p* = 0.5520	*F*(1,35) = 0.8845, *p* = 0.3534
DAT-ph ∼600 kDa	*F*(1,35) = 4.033, *p* = 0.0524	*F*(1,35) = 1.343, *p* = 0.2544	*F*(1,35) = 0.2286, *p* = 0.6356

### CE-104 Modulates Synaptic Dopamine Transporter Levels

**FIGURE 4:**
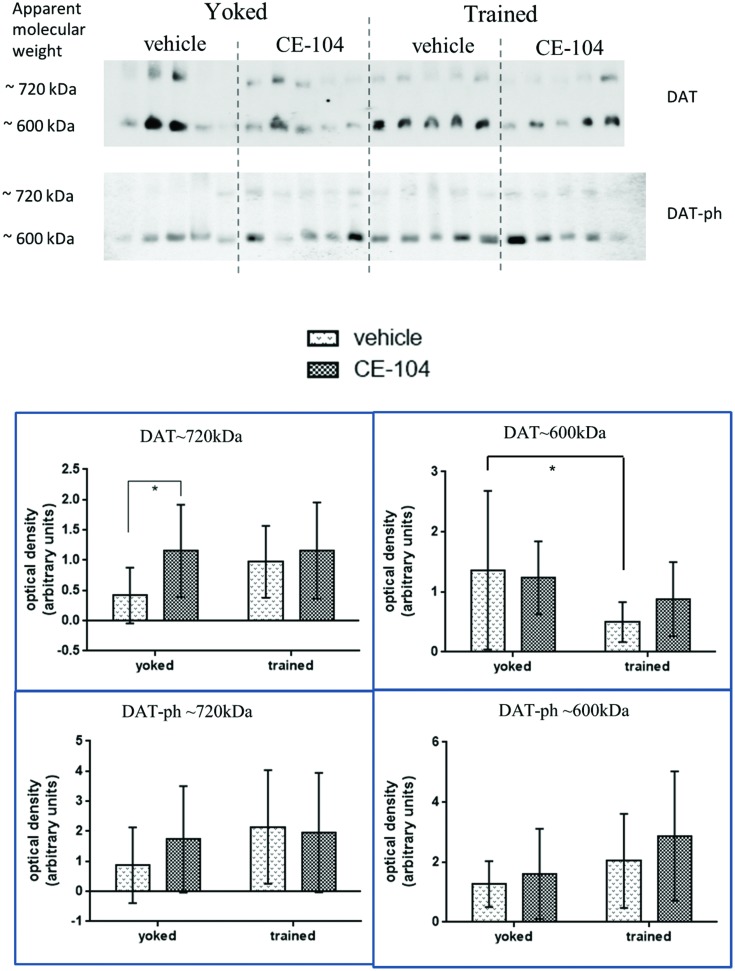
**Representative blot of BN-PAGE Western quantification of dopamine transporter complexes in frontal cortex tissue.** DAT∼720 kDa complex levels were increased due to drug treatment in yoked animals. No differences in phosphorylated DAT Thr^53^ (DAT-ph) complex levels were found. Graph shows mean ± SD, special characters denote level of significance of difference in two-way ANOVA (^∗^*p* < 0.05).

Two-way ANOVA analysis of BN-PAGE Western blot densitometric comparison of DAT complexes were shown in **Figure 4.** For total DAT which was separated at 720 kDa: in the yoked group, DAT∼720 kDa containing complex levels were significantly increased in the drug-treated group compared to vehicle (*p* < 0.05) and in the DAT∼600 kDa containing complex levels were significantly decreased in the trained animals compared to yoked in the vehicle treated groups (*p* < 0.05).

## Discussion

The animals had learned the spatial memory task which is evident from the gradually decreasing WME, RME and latency due to repeated training in the RAM. In order to retrieve the food effectively in the RAM paradigm, rats have to avoid re-visits of arms, which involves WM (Mizumori et al., 1987). Application of CE-104 improved the spatial working but not the reference memory during performance sessions as revealed by less WME in the drug treated rats as compared to controls. Both doses of CE-104 exert similar behavioral effect with no dose-response differences.

From various electrophysiological recordings (Chafee and Goldman-Rakic, 1998), lesioning (Granon et al., 1994; van Asselen et al., 2006), and functional neuroimaging (D’Esposito et al., 2000; Fletcher and Henson, 2001) studies it is evident that the PFC is specifically involved in the processing of WM mechanisms. Low dose psychostimulants generally exert regionally restricted actions elevating extracellular monoamine levels preferentially in the PFC and eventually facilitating neurotransmitter mediated signal processing (Berridge and Devilbiss, 2011). A study revealed that direct infusion of methylphenidate (0.5 μg/500 nl) into the dorsal anterior cingulate/dorsal prelimbic subfields of the medial PFC of rats improves spatial WM performance, comparable to that seen with systemic administration (Spencer et al., 2012). Many such cognitive enhancers inhibit MAT – DAT, SERT, and NET selectively or promiscuously (Berridge and Devilbiss, 2011). The uptake assays revealed that CE-104 blocks DAT rather specifically at 28 μM, suggesting the involvement of dopamine in the improvement of the spatial WM in RAM. Importantly, dopamine afferents within the PFC display a greater sensitivity to a variety of environmental and pharmacologic challenges relative to the subcortical dopamine systems (Roth et al., 1988; Berridge et al., 1999). Other MAT blockers like indeloxazine blocked the reuptake of norepinephrine with an IC_50_ of 3.2 μM (CE-104 at 160 μM) and reuptake of serotonin with an IC_50_ of 0.71 μM (CE-104 negligible effects) and also enhanced the memory in a variety of tests paradigms in the brain regions in a specific monoamine-concentration dependent manner (Yamaguchi et al., 1996).

Measuring monoamine concentrations with *in vivo* microdialysis in rats performing a delayed alternation task in presence of low and clinically relevant doses of methylphenidate revealed increased norepinephrine and dopamine eﬄux within the PFC region but not in the nucleus accumbens and the medial septal area; WM was significantly improved while locomotion and electroencephalographic–electromyographic indices of arousal were only minimally affected (Berridge et al., 2006). This may support the low-dose induced specific enhancement of PFC-dependent cognitive functions without deterioration of the general behavioral organization. The similarity of behavioral effects in the presence of both low doses of CE-104 in our study suggests an appropriate safety margin of CE-104.

We have shown previously that several neurotransmitter RCs rather than receptor subunits, including dopamine receptors are modulated due to training in spatial memory paradigms like multiple T-maze and Barnes maze (Ghafari et al., 2011; Keihan Falsafi et al., 2012). The changes in levels of dopamine RCs and DAT complexes we reported here could be associated with the persistent chemical changes of the neuronal synapses as a result of RAM training and due to the chronic effect of the drug treatment as we did the biochemical analysis after completion of training sessions.

In trained drug-treated animals, D1 receptor containing complexes were significantly elevated when compared to yoked animals suggesting a training induced up-regulation of D1 receptor levels. We can exclude a mere drug induced or a solely training induced modulation since no differences in D1 RCs levels between the related groups could be observed. The involvement of FC D1 receptors in WM mechanism is already well documented. Stimulation of dopamine D1 receptors in the PFC can improve spatial WM related cognitive deficits and may even enhance cognitive function in healthy animals (Mehta and Riedel, 2006). The cognition-enhancing actions CE-104 could be due to the drug-induced enhanced signaling of D1 receptors in the PFC. Consistent with this hypothesis, the ability of low-dose methylphenidate to improve PFC-dependent behavior is hindered by systemically administered D1 antagonists, as measured in delayed-response tasks. These observations infer that the cognition-enhancing effects of low-dose psychostimulants are dependent on D1 receptors located within the PFC (Berridge and Devilbiss, 2011). Additional evidence also suggests a prominent role of PFC D1 receptors in the behavioral and electrophysiological actions of low-dose psychostimulants (Berridge et al., 2012).

The D2 and D3 receptor containing complex levels were down-regulated due to training which could be observed only in vehicle treated group and not in drug treated group. The dopamine D1 but not D3 receptor plays a fundamental role in spatial WM in PFC of mice (Xing et al., 2012), however mice lacking both D2 and D3 receptors have spatial WM deficits (Glickstein et al., 2002). Our results show that the drug down-regulates D2 receptor levels in drug treated animals compared to vehicle group only in untrained animals. These results indicate a differential requirement of D1, D2, and D3 receptor levels in the synapse in mediating the effects of CE-104 on spatial WM and cognitive function.

The DAT is a plasma membrane-spanning protein known to mediate uptake of released dopamine from the extracellular space into the pre-synaptic neuron, thereby maintaining homeostasis. The cell surface expression and its activity are mainly regulated by post-translational modification and binding partner interaction (Vaughan and Foster, 2013). Total DAT complex levels were significantly increased in drug treated groups compared to vehicle in the yoked animals. This signifies that CE-104 not only acts pharmacologically but also alters the DAT levels in the synapse. To support this interpretation, substantial evidence defends that the chronic use of several psychostimulant drug alters the DAT levels as well as its activity, but its mechanisms are not known (Schmitt and Reith, 2010). However, levels of total DAT in trained animals were comparable between CE-104 administered and vehicle treated groups. Regulation of DAT activity by phosphorylation at Thr^53^ plays an important role in modulating its function (Foster et al., 2012). Intriguingly, the levels of phosphorylated DAT were comparable between groups. This could be due to a long-term adaptive response by the dopaminergic system (particularly in synaptic active DAT-ph levels) to the drug induced increase in extracellular dopamine concentrations during learning and plasticity.

CE-104’s ability to inhibit the DAT and its regulation of dopamine receptor and transporter complex levels could be a possible mechanism for its behavioral effects in the RAM. DAT reuptake inhibition is in the order of magnitude as for modafinil and so are cognitive enhancing effects of CE-104 and modafinil at low doses (Karabacak et al., 2015). In addition, the use of more doses are recommended in further studies as modafinil type cognitive enhancers were shown to have diverse and different effects ranging from 0.1 mg/kg body weight up to 120 mg/kg body weight. The cognitive neuromodulators of the DAT inhibition exerts its function in a complex way (Minzenberg and Carter, 2008; Qu et al., 2008; Spiller et al., 2013) and further behavioral, pharmacokinetic, neurotoxicity, and molecular studies in several brain areas are required to reveal the specific mechanistic actions and adverse effects of CE-104.

## Author Contributions

All authors listed, have made substantial, direct and intellectual contribution to the work, and approved it for publication.

## Conflict of Interest Statement

The authors declare that the research was conducted in the absence of any commercial or financial relationships that could be construed as a potential conflict of interest. The reviewer SM and handling Editor declared their shared affiliation, and the handling Editor states that the process nevertheless met the standards of a fair and objective review.
