# Supplementary material for: A Novel Heterocyclic Compound CE-104 Enhances Spatial Working Memory in the Radial Arm Maze in Rats and Modulates the Dopaminergic System
Source: Front Behav Neurosci. 2016 Feb 22;10:20. doi: 10.3389/fnbeh.2016.00020 (PMC4761905; doi:10.3389/fnbeh.2016.00020)
Supplement: Supplementary file 1 [file Data_Sheet_1.DOCX]

## Chemical synthesis of CE-104


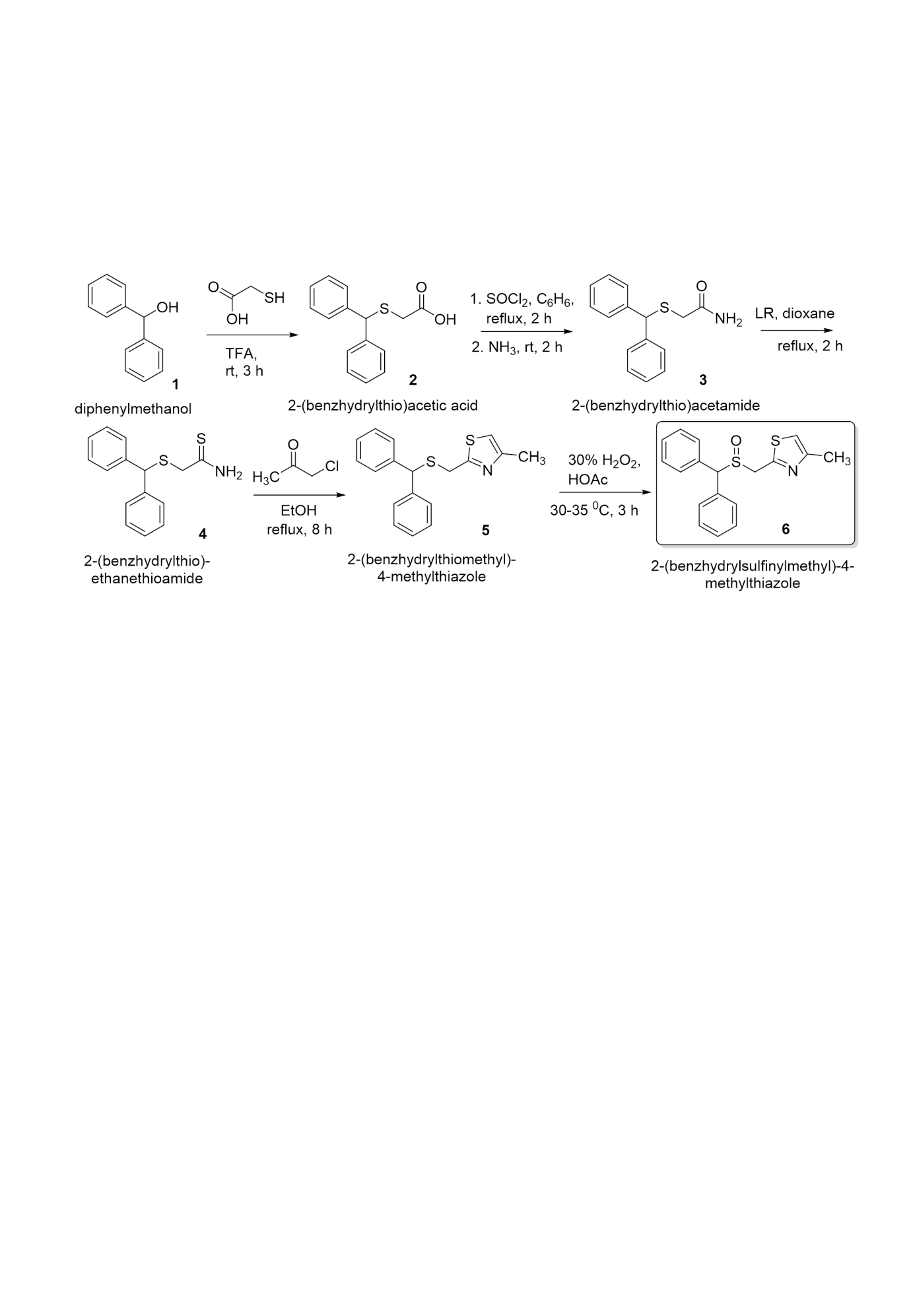


**Supplementary Fig 1** General synthesis scheme of the target 2-(benzhydrylsulfinylmethyl)-4-methylthiazole (CE-104): TFA – Trifluroaceticacid, rt – room temperature, LR - Lawesson’s reagent, EtOH – Ethanol, HOAc – Glacial acetic acid

**2-(Benzhydrylthio)acetic acid (2) synthesis**

Thioglicolic acid (10.50g, 7.92ml (d=1.325), 0.114mol) was added dropwise to the solution of diphenylmethanol (1) in trifluoroacetic acid (100ml). The reaction mixture was stirred at room temperature for 3h. The resulting precipitate was filtered off, washed with water (3 × 50ml) and hexanes. The crude product was recrystallized from ethylacetate/hexanes 1:2 to get a colorless precipitate of acid (2). Yield: 17.93g (64%), melting point (m.p.): 121°C.

NMR ^1^H (DMSO-d_6_+CCl_4_, 400MHz): 2.96 (s, 2H, CH_2_), 5.38 (s, 1H, CH), 7.21 (t, 2H, H_arom._), 7.28-7.30 (t, 4H, H_arom._), 7.40 (d, 4H, H_arom._).

**2-(Benzhydrylthio)acetamide (3) synthesis**

2-(Benzhydrylthio)acetic acid (2) (12.05g, 0466mol) was suspended in anhydrous benzene (60ml) and a solution of SOCl_2_ (22.20g, 13.41ml (d=1.655), 0.186mol) in benzene (14ml) was added to the suspension slowly. The mixture was refluxed for 2h. Then the solvent was evaporated and the resulting oil was re-dissolved in dichloromethane (DCM), cooled in an ice-bath and dry NH_3_ (gas) was bubbled into the solution for 2h. The resulting suspension was diluted with NaHCO_3_ (10% aq. solution) and extracted with DCM. The organic layer was washed with brine and dried over Na_2_SO_4_. The solvent was evaporated and the formed colorless precipitate of amide (3) was washed with ether. Yield: 69% (8.25g), m.p.: 95-100°C.

NMR ^1^H (CDCl_3_, 400 MHz): 3.09 (s, 2H, CH_2_), 5.18 (s, 1H, CH), 5.60 (br. s, 1H, NH), 6.78 (br. s, 1H, NH), 7.25 (t, 2H, H_arom._), 7.32 (t, 4H, H_arom._), 7.40 (d, 4H, H_arom._).

**2-(Benzhydrylthio)ethanethioamide (4) synthesis**

The mixture of amide (3) (4g, 0.015mol) and Lawesson’s reagent (3.44g, 0.0085mol) in anhydrous dioxane (50ml) was refluxed for 2h. The solvent was evaporated till dryness. The resulting yellow oil was dissolved in DCM and washed with water and brine. The solution was dried over Na_2_SO_4_. The obtained crude product was purified with column chromatography (ethylacetate/hexanes 1:2). A pure thioamide (4) was obtained as a colorless precipitate. Yield: 62% (2.63g), m.p.: 96-99°C.

NMR ^1^H (CDCl_3_, 400 MHz): 3.64 (s, 2H, CH_2_), 5.09 (s, 1H, CH), 7.24-7.40 (m, 10H, H_arom._), 7.69 (br. s, 1H, NH), 8.12 (br. s, 1H, NH).

**2-(Benzhydrylthiomethyl)-4-methylthiazole (5) synthesis**

α-chloroacetone (0.41ml, 0.00512mol) was added to the solution of thioamide (4) (1g, 0.00366mol) in dry ethanol (16ml) and the resulting mixture was refluxed for 7h. The solvent was evaporated till dryness and a small amount of ethylacetate was added to the resulting oil. The colorless precipitate obtained was filtered off and washed with ethylacetate to obtain 4-methylthiazole (5). Yield: 76% (0.866g), m.p.: 154°C.

NMR ^1^H (DMSO-d_6_+CCl_4_, 400 MHz): 3.37 (s, 3H, CH_3_), 3.97 (s, 2H, CH_2_), 7.17 (s, 1H, CH_thiazol._), 7.21 (t, 2H, H_arom._), 7.37-7.31 (t, 4H, H_arom._), 7.39 (d, 2H, H_arom._).

MS: [M+1] = 312.08

**2-(Benzhydrylsulfinylmethyl)-4-methylthiazole (6) synthesis**

To the solution of 4-methylthiazole (5) (0.86g, 0.00276mol) in glacial acetic acid (16ml), a solution of H_2_O_2_ (27.6%, d=1.105, 0.31ml) was added. The reaction mixture was stirred at 30-35°C for 5h and subsequently poured on ice. The resulting oil was extracted with ethylacetate and washed with water. The organic layer was evaporated till dryness. The resulting oil was coated with ether. The crude colorless precipitate formed at low temperature was recrystallized from ethylacetate. Yield: 61% (0.553g), m.p.: 104-107°C.

NMR ^1^H (CDCl_3_, 400 MHz): 3.48 (s, 3H, CH_3_), 3.97 (d, 1H, J=13.6 Hz, CH_2_), 4.18 (d, 1H, J=13.6 Hz, CH_2_), 5.13 (s, 1H, CH), (6.89 (s, 1H, CH_thiazol._), 7.32-7.42 (m, 6H, H_arom._), 7.48-7.50 (m, 4H, H_arom._).

MS: [M+1] = 328.08

The final compound (6) was estimated to be more than 95% pure as determined by ^1^H NMR (Proton nuclear magnetic resonance) in deuterated chloroform at 400MHz.
